# Combined Lactoferrin and Osteopontin Treatment Attenuates LPS-Induced Inflammatory Responses and Intestinal Injury in Macrophages and Mice

**DOI:** 10.3390/foods15203746

**Published:** 2026-10-21

**Authors:** Yijia Zhang, Chuangang Li, Wenrui Zhang, Yujuan Chen, Xinyuan Wang, Yifei Zhai, Yanqin Quan, Jian He, Yixuan Li, Yanan Sun

**Affiliations:** 1 College of Food Science and Nutritional Engineering, China Agricultural University, Beijing 100083, China; lxzyj001@163.com; 2 Key Laboratory of Precision Nutrition and Food Quality, Department of Nutrition and Health, China Agricultural University, Beijing 100193, China; yifei0109@163.com (C.L.); wrzhang2021@163.com (W.Z.); chenyujuan2000@163.com (Y.C.); 15022112227@163.com (X.W.); zyifei1f@163.com (Y.Z.); 15135988211@163.com (Y.Q.); syn612@cau.edu.cn (Y.S.); 3 National Center of Technology Innovation for Dairy, Hohhot 010000, China

**Keywords:** lactoferrin, osteopontin, LPS-induced intestinal injury, intestinal barrier

## Abstract

This study explored the combined anti-inflammatory potential of lactoferrin (LF) and osteopontin (OPN) using both in vitro (LPS-challenged macrophages) and in vivo (LPS-induced intestinal injury) models. The LF-OPN combination demonstrated superior efficacy over monotherapies in suppressing pro-inflammatory mediator expression in macrophages. In vivo, co-administration markedly attenuated intestinal-barrier disruption and preserved mucosal architecture, evidenced by increased villus height, crypt depth, and goblet-cell counts, surpassing the effects of OPN alone. This structural protection was accompanied by significant immunomodulation; the combination treatment elevated systemic and local anti-inflammatory cytokines while reducing pro-inflammatory ones more effectively than either single protein. Notably, at LF:OPN ratios of 1:1, 5:1, and 10:1, murine intestinal *Il10* concentrations rose by 44.25%, 54.46%, and 60.02%, respectively, relative to LF group; *Tnfα* concentrations fell by 33.73%, 52.27%, and 37.80% at the same three ratios when benchmarked against the OPN group (all *p* < 0.05). These data reveal a potent combined effect of LF and OPN against acute inflammatory insults, suggesting a promising therapeutic combined effect. Future work should focus on elucidating the underlying signaling mechanisms and evaluating efficacy in chronic disease models.

## 1. Introduction

An intact intestinal epithelial barrier is indispensable for maintaining intestinal homeostasis [1]. As a major component of the outer membrane of Gram-negative bacteria, lipopolysaccharide (LPS) can initiate pro-inflammatory signaling following recognition by host pattern-recognition receptors and compromise epithelial barrier integrity, ultimately contributing to LPS-induced intestinal injury. LPS-induced intestinal injury is commonly characterized by increased intestinal permeability, altered epithelial morphology, disruption of tight-junction integrity, and upregulation of inflammatory mediator expression [2,3]. Barrier dysfunction may further facilitate the translocation of LPS, bacterial components, and other luminal antigens into the lamina propria, portal circulation, or systemic circulation, thereby amplifying local inflammation and potentially contributing to endotoxemia and low-grade systemic inflammation [4,5]. Persistent or recurrent impairment of intestinal-barrier function and gut-derived LPS translocation have been associated with adverse health outcomes, including metabolic dysfunction, fatty liver disease, and elevated cardiovascular risk [6]. Therefore, mitigating LPS-induced intestinal injury and its associated adverse effects is important for maintaining intestinal and systemic health.

Lactoferrin (LF) is an iron-binding glycoprotein endowed with diverse biological activities. This protein is widely distributed in biological secretions and is present at particularly high concentrations in human, bovine, and other mammalian milk [7]. As a naturally occurring bioactive molecule, LF exerts broad-spectrum antibacterial activity through iron chelation and disruption of bacterial membrane integrity; it also inhibits viral attachment and replication while inducing antiviral immune responses [8]. Additionally, LF modulates inflammatory signaling, suppresses pro-inflammatory cytokine production, and promotes the release of anti-inflammatory mediators. These actions confer broad anti-inflammatory and immunomodulatory properties, including the modulation of leukocyte activity and maintenance of immune homeostasis [9]. LF upregulates the expression of tight-junction proteins, promotes intestinal stem-cell proliferation and differentiation, enhances goblet-cell mucus secretion, preserves intestinal-barrier integrity, and inhibits endotoxin translocation and bacterial translocation [10]. Additionally, LF possesses antioxidant and osteogenic activities, as well as metabolic-regulatory and iron-absorption-enhancing properties [11]. Osteopontin (OPN) is a multifunctional glycoprotein detected in various tissues, including bone, liver, intestine, and mammary gland, and is also present in biological fluids such as milk, blood, saliva, and others. Multiple cell types contribute to its production, notably osteoblasts, macrophages, T lymphocytes, and intestinal epithelial cells [12]. OPN maintains skeletal integrity by regulating bone mineralization, osteoblast–osteoclast adhesion, and bone remodeling. In the intestine, OPN promotes epithelial cell proliferation and differentiation, increases villus height and crypt depth, enhances goblet-cell mucus secretion, preserves intestinal-barrier integrity, and maintains intestinal immune homeostasis [13]. Within the immune system, OPN serves as a key immunomodulatory mediator that activates macrophages, T cells, and NK cells; modulates the balance between pro-inflammatory and anti-inflammatory cytokines; and participates in the regulation of inflammation, pathogen clearance, and maintenance of immune tolerance [14]. In the context of neurodevelopment, OPN has been implicated in neuronal growth and myelination, cognitive function, and protection against ischemic brain injury [15,16]. Moreover, OPN contributes to tissue repair processes such as wound healing and angiogenesis, while also regulating glucose and lipid metabolism and improving insulin sensitivity, thereby contributing to systemic metabolic homeostasis [17].

Accumulating evidence indicates that both LF and OPN can ameliorate intestinal-barrier dysfunction. A recent study by Li et al. demonstrated that co-administration of LF and OPN at an LF:OPN ratio of 1:5 exerted a protective effect against LPS-induced intestinal injury in mice, primarily by enhancing intestinal stem-cell proliferation and the differentiation of absorptive epithelial cells via the Notch–HES1 pathway, accompanied by restoration of tight-junction protein expression and reduction of intestinal permeability [18]. Although previous studies have established the barrier-protective effects of LF-OPN combinations, such investigations remain limited to architectural and restorative features of the gut lining. The present study seeks to explore whether LF and OPN exert combined anti-inflammatory effects using complementary in vitro and in vivo experimental models. Specifically, we examined their capacity to attenuate LPS-induced inflammatory activation in macrophages and LPS-induced intestinal injury in mice.

## 2. Materials and Methods

### 2.1. Materials and Reagents

Hilmar Ingredients (Hilmar, CA, USA) provided bovine lactoferrin (LF; >95% pure, 10.5% iron-saturated). Arla Foods Ingredients (Aarhus, Videbæk, Denmark) made available bovine osteopontin (OPN; >95% pure). Lipopolysaccharide (LPS) was acquired from Shanghai Yuanye Bio-Technology Co., Ltd. (Shanghai, China).

### 2.2. Cell Culture and Model Establishment

RAW264.7 cells, a murine macrophage line obtained from the American Type Culture Collection (ATCC), were cultured in Dulbecco’s modified Eagle’s medium (DMEM) supplemented with 10% fetal bovine serum (FBS) plus 1% penicillin–streptomycin. Cultures were maintained at 37 °C under humidified conditions with 5% CO_2_. At the time of treatment, RAW264.7 cells were randomly assigned to seven experimental groups. The control group received no treatment. Cells in the LPS condition received 1 μg/mL LPS alone. For the LF and OPN conditions, cells were co-treated with 1 μg/mL LPS and either 1 mg/mL LF or 1 mg/mL OPN. Three additional co-treatment arms employed LF:OPN blends at ratios of 1:1, 5:1, and 10:1. The corresponding protein concentrations were (500 μg/mL LF + 500 μg/mL OPN), (833.33 μg/mL LF + 166.67 μg/mL OPN), and (909.09 μg/mL LF + 90.91 μg/mL OPN), respectively, with 1 μg/mL LPS added to all three conditions. Treatments began when cultures reached 80% confluence and continued over a 24 h period; samples were then collected for downstream analysis.

### 2.3. Cell Viability Assay

After treatment, cellular viability was assessed using the CCK-8 colorimetric assay. Per the supplier’s instructions, CCK-8 solution was dispensed into individual wells, followed by a 60 min of incubation at 37 °C. Absorbance at 450 nm was then recorded using a microplate reader.

### 2.4. Animal and Diet

Forty-two 4-week-old male C57BL/6N mice were purchased from Vital River Laboratory Animal Technology Co. Ltd. (Beijing, China). Mice were housed in SPF-grade barrier facilities within the Laboratory Animal Platform at China Agricultural University. The housing environment was kept at 20–24 °C and 55–60% humidity, with alternating 12 h periods of light and darkness. Regular rodent diet and drinking water were available ad libitum throughout the study. All experimental procedures were conducted in accordance with institutionally approved animal welfare guidelines. The Animal Welfare and Animal Experiment Ethics Committee of China Agricultural University issued ethical approval (permit no. AW09232202-5-1).

Forty-two male C57BL/6N mice aged 4 weeks were allowed to acclimate for 7 days prior to random assignment to seven experimental groups, each comprising 6 animals: control, LPS, LF, OPN, LF:OPN = 1:1, LF:OPN = 5:1, and LF:OPN = 10:1. The interventions were administered by daily intragastric gavage. The LF and OPN groups each received 300 mg/kg BW of their respective protein. Three co-treatment arms received LF:OPN blends at 1:1, 5:1, and 10:1 ratios, corresponding to (150 mg/kg BW LF + 150 mg/kg BW OPN), (250 mg/kg BW LF + 50 mg/kg BW OPN), and (272.73 mg/kg BW LF + 27.27 mg/kg BW OPN), respectively. Animals assigned to control and LPS conditions received sterile saline in volumes equivalent to those used for treated animals. Following 7 days of daily intragastric administration, all groups except the control group were challenged by intraperitoneal injection of 200 μg LPS; control animals were given sterile saline instead. Animals were sacrificed at 24 h following LPS challenge, and tissues were harvested for analysis. Body mass and food consumption were documented each day.

### 2.5. Animal Sample Collection

Animals underwent a 12 h overnight fast with free access to drinking water. Blood was subsequently collected from the orbital venous plexus. Mice were then euthanized through cervical dislocation. The obtained blood specimens were left to coagulate and thereafter centrifuged at ambient temperature. The upper serum layer was aspirated and kept frozen at −80 °C. Spleens were excised and weighed individually. Two jejunal segments (3–5 cm per segment) were collected: one segment was immersed in tissue fixative for histological analysis, whereas the other was immediately snap-frozen in liquid nitrogen and stored until further analysis.

### 2.6. Intestinal Permeability Assay

The jejunal segment was ligated at both ends and injected with fluorescein isothiocyanate (FITC)-labeled dextran. Following a 37 °C water-bath incubation step, gut permeability was evaluated via fluorometric quantitation on a microplate reader.

### 2.7. Hematoxylin–Eosin Staining

The fixed jejunal specimens were first subjected to dehydration, paraffin embedding, and sectioning. Thereafter, slides were deparaffinized in xylene, followed by rehydration through a graded ethanol series of decreasing concentrations. Nuclei were stained with hematoxylin for 30 s. Eosin was then used as a counterstain for 1 min. Once dehydrated, the slides were coverslipped. Jejunal morphology was assessed under a light microscope. We subsequently measured villus height, crypt depth, and the villus-to-crypt ratio, followed by statistical analysis of these data.

### 2.8. Alcian Blue Staining

Jejunal tissues, after fixation, underwent sequential dehydration, paraffin embedding, and sectioning. Following xylene deparaffinization and rehydration in a graded ethanol series, slides were immersed in acidified Alcian Blue for 10 min. Slides were next placed into Alcian Blue solution and maintained at ambient temperature for 30 min. Nuclear Fast Red was subsequently applied for counterstaining and incubated for 10 min. Thereafter, slides underwent dehydration, clearing, and mounting. Photomicrographs were captured, and the average goblet-cell count per villus was calculated.

### 2.9. PAS Staining

Once fixed, jejunal specimens were processed through dehydration, paraffin infiltration, and microtome sectioning. Following xylene dewaxing and rehydration in a graded ethanol series, slides underwent a sequential staining protocol. The steps included hematoxylin (5–10 min), periodic acid oxidation (5 min), Schiff’s reagent (10 min), and sodium metabisulfite (1 min). Sections were then dehydrated, cleared, and mounted. Micrographs were captured, and the mean number of secretory cells per villus was quantified.

### 2.10. RT-qPCR Analysis

TRIzol reagent was used to extract total RNA from both cultured cell samples and jejunal tissue specimens. Reverse transcription was then performed with the All-In-One 5× RT MasterMix kit for cDNA synthesis. Transcript levels of selected genes were measured and quantified by reverse-transcription quantitative polymerase chain reaction (RT-qPCR). The targeted genes included *Toll-like receptor 4* (*Tlr4*); *C-C motif chemokine ligands 2* and *C-C motif chemokine ligands 3* (*Ccl2* and *Ccl3*); *myeloid differentiation primary response 88* (*Myd88*); *tumor necrosis factor alpha* (*Tnfα*); and *interleukin 4, 10, 1alpha, 1beta*, and *interleukin 6* (*Il4*, *Il10*, *Il1a*, *Il1b*, and *Il6*). *Gapdh* was used as the reference gene to normalize relative transcript levels across all cellular and tissue specimens. All primer pairs are detailed in Table 1.

### 2.11. ELISA

Commercially available ELISA kits were employed to quantify cytokine concentrations. Specifically, levels of IL-1β, IL-4, IL-6, IL-10, and TNF-α were measured in cell lysates, serum, and jejunal tissue homogenates following protocols supplied by each manufacturer.

### 2.12. Statistical Analysis

Statistical analyses were conducted using SPSS version 20.0 and GraphPad Prism 10. Results are expressed as mean ± standard deviation. The Shapiro–Wilk method first assessed data normality, and Levene’s procedure examined homogeneity of variance. One-way ANOVA was subsequently employed, and Tukey’s HSD post hoc analysis was utilized for comparisons among groups. A *p*-value below 0.05 was considered statistically significant.

## 3. Results

### 3.1. Effects of LPS on Macrophage Viability and Inflammatory Cytokine Expression

To determine the appropriate concentration of LPS for macrophage stimulation, cell viability was first assessed across a range of LPS concentrations to identify a non-cytotoxic concentration. We subsequently used RT-qPCR to examine whether these concentrations altered the expression of inflammatory genes in macrophages, thereby identifying the optimal stimulation conditions. Both 1 and 5 μg/mL LPS had no significant cytotoxic effects, with cell viability values of 89.78% and 81.38%, respectively; neither value differed significantly from that of the control group (*p* > 0.05) (Figure 1A). Compared with untreated cells, stimulation with 1 μg/mL LPS markedly increased levels of *Tlr4*, *Myd88*, *Ccl2*, *Ccl3*, *Il1a*, *Il1b*, *Il6*, and *Tnfa* (*p* < 0.05), while concurrently reducing *Il10* mRNA abundance (*p* < 0.05) (Figure 1B–J). Exposure to 5 μg/mL LPS likewise elevated *Ccl3*, *Il1a*, *Il1b*, *Il6*, and *Tnfa* transcripts (*p* < 0.05; Figure 1E–I), but diminished *Il10* mRNA amounts (*p* < 0.05; Figure 1J). In contrast, the mRNA levels of *Tlr4*, *Myd88*, and *Ccl2* did not differ significantly from those in untreated cells (*p* > 0.05) (Figure 1B–D). Importantly, cells receiving 1 μg/mL LPS showed greater induction of *Tlr4*, *Myd88*, *Il1a*, *Il6*, and *Tnfa* compared with those given 5 μg/mL LPS (*p* < 0.05) (Figure 1B,C,F,H,I), corresponding to elevations of 33.36%, 25.66%, 59.46%, 46.86%, and 33.35%. Conversely, *Il10* transcript abundance was reduced by 52.87% (*p* < 0.05). Collectively, these results suggest that 1 μg/mL LPS induced a more pronounced inflammatory response than the 5 μg/mL dose; Therefore, 1 μg/mL was selected as the LPS concentration for all subsequent cellular experiments.

### 3.2. LF and OPN Dosages and Proportions Modulate Macrophage Survival and Pro-Inflammatory Mediator Transcript Abundance

To determine the intervention concentrations of LF and OPN, the effects of different concentrations of LF and OPN on macrophage viability were first assessed. We next assessed whether different LF:OPN ratios differentially regulated the expression of inflammatory genes in macrophages following protein treatment. Compared with untreated cells, exposure to LF or OPN at doses ≥ 5 mg/mL significantly reduced cell viability (*p* < 0.05), whereas concentrations ≤ 1 mg/mL did not produce a notable change in viability (*p* > 0.05). Accordingly, the intervention concentration for both LF and OPN was set at 1 mg/mL (Figure 2A).

Subsequently, macrophages were treated with LF and OPN composite proteins at ratios of 1:1, 5:1, and 10:1 (LF:OPN). Compared with untreated controls, LPS stimulation markedly increased transcript abundance of *Tlr4*, *Ccl2*, *Ccl3*, *Il1a*, *Il1b*, *Il6*, and *Tnfa* (*p* < 0.05) (Figure 2B–H), while significantly suppressing *Il10* mRNA levels (*p* < 0.05) (Figure 2I). Both LF and OPN monotherapies reduced levels of *Tlr4*, *Ccl2*, *Ccl3*, *Il1a*, *Il1b*, *Il6*, and *Tnfa* relative to the LPS group (*p* < 0.05) (Figure 2B–H), and elevated *Il10* transcript amounts (*p* < 0.05) (Figure 2I). All LF–OPN combination regimens diminished *Tlr4* and *Il1b* transcript abundance compared with LF alone (*p* < 0.05) (Figure 2B,F). Additionally, the 5:1 and 10:1 LF-to-OPN ratios also suppressed *Ccl2*, *Ccl3*, *Il1a*, *Il6*, and *Tnfa* compared to the LF group (*p* < 0.05) (Figure 2C–E,G,H). Compared with OPN monotherapy, all LF–OPN combination groups exhibited significantly lower mRNA levels of *Tlr4*, *Ccl3*, *Il1a*, *Il1b*, *Il6*, and *Tnfa* (*p* < 0.05) (Figure 2B,D–H). The 5:1 LF:OPN ratio further decreased *Ccl2* transcript levels (*p* < 0.05) (Figure 2C). Remarkably, *Il10* expression in the LF:OPN = 1:1 and 5:1 combination groups was significantly elevated compared with either the LF or OPN group (*p* < 0.05; Figure 2I).

### 3.3. Different LF and OPN Combinations Alter Inflammatory-Factor Protein Levels Within Macrophages

We next investigated whether different LF:OPN ratios differentially affected anti-inflammatory response by measuring inflammatory cytokine protein levels in macrophage lysates following protein treatment. Compared with untreated cells, LPS stimulation significantly reduced IL-4 and IL-10 protein amounts (*p* < 0.05) (Figure 3A,B), while significantly increasing IL-6, IL-1β, and TNF-α protein levels (*p* < 0.05) (Figure 3C–E). All protein-treatment groups exhibited significantly higher IL-4 and IL-10 protein concentrations than the LPS group (*p* < 0.05; Figure 3A,B), whereas IL-1β, IL-6, and TNF-α protein levels were significantly reduced (*p* < 0.05) (Figure 3C–E). Importantly, all LF–OPN combination regimens resulted in significantly higher IL-10 protein abundance than LF monotherapy (*p* < 0.05) (Figure 3B). The 5:1 and 10:1 LF-to-OPN ratios also produced significantly higher IL-10 protein levels than OPN monotherapy (*p* < 0.05) (Figure 3B). The 5:1 LF-to-OPN ratio significantly elevated IL-4 protein levels compared with either single-protein treatment (*p* < 0.05) (Figure 3A). All LF–OPN combination groups exhibited significantly lower IL-1β, IL-6, and TNF-α protein levels when compared with LF or OPN monotherapy (*p* < 0.05; Figure 3C–E), with the most pronounced reduction observed for IL-6 (Figure 3D). Specifically, compared with LF monotherapy, IL-6 protein expression in LF:OPN = 1:1, 5:1, and 10:1 groups was reduced by 14.13%, 40.22%, and 25.78%, respectively, and by 26.58%, 48.41%, and 35.95%, respectively, relative to the OPN group (Figure 3D).

### 3.4. Different LF and OPN Combinations Influence Body Mass and Splenic Index in a Murine Model

To investigate the effects of composite proteins at different ratios on basic physiological parameters in mice, changes in body weight during the intervention period and spleen indices after treatment were recorded. All groups exhibited a gradual increase in body weight during the intragastric administration period. Eight days after the experiment began, after LPS administration, all LPS-challenged groups exhibited a significant reduction in body weight compared with the control group (*p* < 0.05). At the same time point, animals receiving protein treatment exceeded the LPS group in body weight (*p* < 0.05). Importantly, the 1:1 and 5:1 LF-to-OPN ratios resulted in significantly higher body weights than either LF or OPN monotherapy (*p* < 0.05) (Figure 4A). LPS challenge substantially elevated spleen index relative to control group (*p* < 0.05). Conversely, both LF monotherapy and all LF–OPN combination regimens markedly decreased spleen index compared with the LPS group (*p* < 0.05). Within the combination-treatment groups, the 5:1 LF-to-OPN ratio achieved the lowest spleen index, surpassing either single-protein treatment (*p* < 0.05), corresponding to decreases of 16.64% and 22.54% (Figure 4B).

### 3.5. Different LF and OPN Combinations Modulate Gut-Barrier Permeability and Mucosal Architecture in a Murine Model

We assessed intestinal-barrier integrity and permeability across all experimental conditions by measuring intestinal permeability, H&E histopathological scores, villus height, and crypt depth. Relative to control group, LPS challenge caused a marked increase in intestinal permeability (*p* < 0.05). All protein intervention groups showed significantly lower intestinal permeability than the LPS group (*p* < 0.05). All composite protein intervention groups displayed significantly lower intestinal permeability than LF group (*p* < 0.05). Importantly, the 5:1 LF-to-OPN ratio diminished gut permeability more effectively than either single-protein treatment (*p* < 0.05), with values remaining comparable to untreated controls (*p* > 0.05; Figure 5B).

LPS stimulation produced substantially higher H&E histopathological scores than the control group (*p* < 0.05), alongside reduced villus height, shallower crypts, and a lower villus-to-crypt ratio (*p* < 0.05). In contrast, all protein-treatment groups showed lower histopathological scores, greater villus height, and an elevated villus-to-crypt ratio than the LPS group (*p* < 0.05; Figure 5C–F). With the exception of OPN monotherapy, all remaining protein-treatment groups displayed deeper crypts than the LPS group (*p* < 0.05) (Figure 5E). Villus-to-crypt ratios did not differ notably across various protein-treated cohorts (*p* > 0.05) (Figure 5F). The LF:OPN = 5:1 and 10:1 groups showed significantly lower histopathological scores than the OPN group (*p* < 0.05), with reductions of 26.69% and 29.12%, respectively (Figure 5C). All composite protein intervention groups exhibited significantly greater villus length and crypt depth than OPN group (*p* < 0.05) (Figure 5D,E). The 5:1 and 10:1 LF–OPN blended protein regimens produced substantially deeper crypts versus LF monotherapy (*p* < 0.05), rising by 30.66% and 30.32%, correspondingly (Figure 5E).

### 3.6. Different LF and OPN Combinations Enhance Goblet-Cell Enumeration in Murine Intestinal Tissue

We assessed mucosal barrier integrity among all study groups by quantifying goblet-cell abundance (Figure 6A,B). Compared with the control group, LPS challenge significantly reduced the number of goblet cells (*p* < 0.05). All protein-treatment groups showed significantly higher goblet-cell counts than LPS group (*p* < 0.05). Notably, all composite protein intervention groups displayed significantly higher goblet-cell counts than OPN group (*p* < 0.05), with increases of 12.54%, 17.36%, and 15.50%, respectively. Goblet-cell enumeration was markedly greater in the 5:1 and 10:1 LF–OPN combination cohorts relative to LF alone (*p* < 0.05), corresponding to elevations of 9.45% and 7.72%, respectively (Figure 6B).

### 3.7. Different LF and OPN Combinations Boost Secretory-Cell Abundance in Murine Gut Mucosa

We next examined intestinal-barrier performance and immune status across the various experimental groups by enumerating secretory-cell counts within murine intestinal tissue (Figure 7A,B). When compared with the control group, LPS administration substantially lowered secretory-cell abundance (*p* < 0.05). All protein intervention groups showed significantly higher secretory-cell counts than the LPS group (*p* < 0.05). All LF–OPN combination groups displayed significantly higher secretory-cell counts than the OPN group (*p* < 0.05), with increases of 18.60%, 20.01%, and 20.83%, respectively. We observed markedly elevated secretory-cell numbers in the 5:1 and 10:1 LF–OPN groups compared with LF group (*p* < 0.05), reflecting gains of 17.07% and 17.87%, respectively (Figure 7B).

### 3.8. Different LF and OPN Combinations Attenuate Circulating Inflammatory Mediator Concentrations in a Murine Model

To compare the anti-inflammatory effects of different groups, serum inflammatory cytokine expression levels were determined. Compared with the control group, LPS challenge markedly reduced circulating IL-4 and IL-10 levels (*p* < 0.05) (Figure 8A,B), while concurrently increasing IL-1β, IL-6, and TNF-α amounts (*p* < 0.05) (Figure 8C–E). Every protein-supplemented cohort showed increased circulating IL-4 and IL-10 levels compared with the LPS group (*p* < 0.05; Figure 8A,B), while IL-1β, IL-6, and TNF-α protein amounts were concurrently diminished (*p* < 0.05; Figure 8C–E). Importantly, all LF–OPN combination regimens produced greater IL-4 and IL-10 concentrations than either single-protein treatment (*p* < 0.05) (Figure 8A,B). The combination groups also showed diminished IL-1β, IL-6, and TNF-α amounts relative to the LF or OPN group (*p* < 0.05) (Figure 8C–E). No significant differences in IL-4, IL-10, IL-1β, IL-6, or TNF-α were detected among the different LF–OPN ratio groups (*p* > 0.05). Overall, the composite protein intervention groups demonstrated superior anti-inflammatory effects compared to LF or OPN monotherapy.

### 3.9. Different LF and OPN Combinations Dampen Gut Mucosal Inflammatory Mediator Transcript Levels Within Murine Intestine

We next evaluated whether different treatments mitigated gut inflammation by measuring inflammatory cytokine transcript levels within intestinal tissue. LPS challenge substantially reduced *Il10* transcript abundance compared with control group (*p* < 0.05; Figure 9A), while concurrently elevating *Tnfa*, *Il6*, and *Il1b* concentrations (*p* < 0.05; Figure 9B–D). All protein-treatment groups exhibited significantly higher intestinal *Il10* transcript levels relative to LPS group (*p* < 0.05). Among these groups, all LF–OPN combination regimens surpassed LF group in *Il10* mRNA levels (*p* < 0.05) (Figure 9A). All protein-treated groups showed diminished intestinal transcript abundance of *Il1b*, *Il6*, and *Tnfa* when benchmarked against LPS group (*p* < 0.05). The 1:1 and 5:1 LF-to-OPN ratios achieved the greatest reduction in *Il1b* transcript levels, outperforming both LF and OPN monotherapy (Figure 9B). Additionally, all LF–OPN combination regimens showed substantially lower *Tnfa* concentrations relative to either single-protein treatment (*p* < 0.05; Figure 9D). No significant differences in *Il10*, *Il1b*, and *Il6* mRNA abundance were observed among the different LF–OPN combination groups (*p* > 0.05; Figure 9A–C).

## 4. Discussion

The intestine, as a major organ involved in digestion, nutrient absorption, barrier defense, and immune regulation in the body, performs essential functions, including nutrient digestion and absorption, prevention of pathogen invasion, maintenance of immune homeostasis, and regulation of gut microbial homeostasis, making it a key organ for maintaining systemic metabolism, immunity, and physiological homeostasis [19]. As key effector cells of the intestinal innate immune system, macrophages play a critical role in regulating intestinal inflammation. Dysregulated macrophage activity is an important contributor to the disruption of intestinal immune homeostasis and the persistence of chronic inflammation. Under physiological conditions, intestinal macrophages generally exhibit an inflammation-resolving or anti-inflammatory phenotype. These cells sustain immune tolerance, modulate microbial equilibrium, and contribute to mucosal healing through release of anti-inflammatory mediators, including IL-10 and transforming growth factor-β (TGF-β), thereby contributing to the maintenance of intestinal-barrier integrity [20]. Conversely, under pathological setting of intestinal inflammation, macrophages are driven to differentiate into the pro-inflammatory M1 subtype. These M1-polarized macrophages secrete large quantities of pro-inflammatory mediators such as TNF-α, IL-1β, and IL-6, which in turn activate inflammatory signaling cascades; promote local intestinal inflammation; damage intestinal epithelial cells; and exacerbate villus atrophy, crypt architectural disruption, and mucus barrier dysfunction [21]. In the present work, macrophages of LPS group exhibited significantly increased transcript levels of multiple pro-inflammatory genes compared with those in the control group (*p* < 0.05), together with a significant reduction in *Il10* transcript levels (*p* < 0.05). These patterns suggest that LPS promotes macrophage polarization toward a pro-inflammatory M1 state and concurrently suppresses conversion to the anti-inflammatory M2 lineage. This observation aligns with the findings previously reported by Shi et al. [19]. A study by Hong et al. showed that lactoferrin (LF) is capable of modulating macrophage polarization in favor of the anti-inflammatory M2 phenotype, inhibiting M1 pro-inflammatory macrophage activation, and alleviating chronic inflammatory persistence [22]. Joung et al., using an early nutritional intervention model, found that OPN promotes M2 anti-inflammatory macrophage polarization and suppresses M1 pro-inflammatory macrophage activation, thereby contributing to the restoration of immune homeostasis [15]. Consistent with previous studies, single-agent treatment with either LF or OPN substantially diminished mRNA levels of *Tlr4*, *Ccl2*, *Ccl3*, *Il1a*, *Il1b*, *Il6*, and *Tnfa* compared with LPS stimulation alone (*p* < 0.05). These treatments simultaneously increased *Il10* transcript amounts (*p* < 0.05). Notably, co-administration of the two proteins produced even greater reduction in *Tlr4* and *Il1b* mRNA expression than LF monotherapy (*p* < 0.05). Similarly, when compared with OPN alone, the blended-protein cohorts showed substantial reductions in *Tlr4*, *Ccl3*, *Il1a*, *Il1b*, *Il6*, and *Tnfa* expression (*p* < 0.05). Together, these observations support the utility of LF–OPN co-treatment to attenuate macrophage-associated inflammatory responses.

Our previous study evaluated a broader LF:OPN ratio range in an LPS-induced mouse intestinal injury model and identified LF:OPN = 1:5 as the most effective ratio for the specific permeability-related outcomes assessed, including serum DAO activity, D-lactate, and FITC–dextran permeability. That study subsequently focused on intestinal epithelial proliferation and differentiation, including the involvement of HES1/Notch-associated regulatory mechanisms [18]. In contrast, the present study examined LF-enriched ratios (1:1, 5:1, and 10:1) and emphasized inflammatory outcomes in LPS-stimulated macrophages and in mice with an LPS-induced intestinal injury model. The two studies used independent animal cohorts and were conducted under the same institutional ethics approval.

Intestinal permeability is a core indicator of intestinal-barrier integrity and physiological homeostasis. FITC, as a commonly used tracer, accurately reflects changes in intestinal permeability, and its levels are directly associated with barrier function strength and intestinal-health status [23]. In healthy physiological states, gut epithelial tight junctions preserve their structural integrity, while gut permeability stays low. This arrangement permits selective nutrient absorption and helps preserve mucosal immune tolerance [19]. When intestinal permeability is abnormally elevated, it leads to structural disruption of the intestinal barrier, directly triggering chronic local intestinal inflammation, inducing massive release of pro-inflammatory cytokines, impairing both physical and immune barrier functions, and promoting the onset and progression of intestinal diseases [19]. Park et al. found that LPS increases intestinal permeability [24]. Similarly, we found that the LPS group exhibited significantly elevated intestinal permeability (*p* < 0.05). Das et al. reported that gut-derived OPN significantly reduces intestinal permeability and maintains intestinal-barrier integrity [13]. Rizzi et al. found that LF can decrease intestinal permeability [25]. In line with these findings, LF and OPN monotherapies both attenuated gut-barrier leakage relative to the LPS group (*p* < 0.05). All LF–OPN combination regimens produced a pronounced reduction in intestinal permeability compared to the LF group (*p* < 0.05). This finding indicates that LF–OPN co-administration provides an enhanced protective effect on intestinal-barrier function in the context of LPS-induced intestinal injury.

The intestinal mucosa comprises multiple specialized cell populations, including epithelial cells, goblet cells, enteroendocrine cells, Paneth cells, and intestinal stem cells, and its structural integrity directly is essential for normal intestinal functions [4]. Villus length is a core indicator of intestinal absorptive surface area: longer villi provide a greater nutrient absorption surface and higher nutrient transport efficiency, whereas villus shortening leads to mucosal atrophy and impaired absorptive function, commonly observed in inflammatory, malnourished, or infected states [4]. Both LF and OPN treatments significantly increased villus height relative to LPS group (*p* < 0.05). Moreover, all LF–OPN combination groups exhibited significantly greater villus height than OPN group (*p* < 0.05). Crypt depth reflects intestinal stem-cell proliferative activity and epithelial renewal capacity: deeper crypts indicate greater epithelial proliferative and regenerative potential, whereas crypt shortening or shallowing may be associated with impaired epithelial renewal and mucosal repair under inflammatory conditions [4]. The results showed that, except for OPN group, all other protein intervention groups had significantly greater crypt depth than LPS group (*p* < 0.05), and all LF–OPN combination regimen produced markedly deeper crypts than OPN monotherapy (*p* < 0.05). The villus length-to-crypt depth ratio is a comprehensive indicator for evaluating overall intestinal development and functional maturation: a higher ratio generally reflects better-preserved intestinal architecture and absorptive capacity, whereas a reduced ratio suggests intestinal dysplasia or inflammatory damage [4]. In this study, the data revealed that the villus-to-crypt ratio across all protein-treated cohorts was markedly elevated relative to the LPS group (*p* < 0.05). The abundance of goblet cells dictates mucus layer thickness and barrier integrity. Through mucin secretion, goblet cells form a protective mucus layer that limits microbial adhesion, exposure to luminal toxins, and penetration of potentially harmful substances into the epithelium. A reduction in goblet-cell abundance can disrupt the mucus barrier, increase intestinal permeability, and enhance the risk of bacterial translocation, representing an important pathological feature of intestinal injury [26]. We observed that goblet-cell abundance was elevated across all protein-treated cohorts relative to the LPS group (*p* < 0.05). In particular, all LF–OPN combination regimens resulted in a greater number of goblet cells than OPN monotherapy (*p* < 0.05). Moreover, the 5:1 and 10:1 LF-to-OPN ratios also produced significantly higher goblet-cell abundance than LF monotherapy (*p* < 0.05). Secretory cells (including enteroendocrine cells, Paneth cells, etc.) are responsible for secreting antimicrobial peptides, digestive enzymes, hormones, and cytokines. Paneth cells secrete defensins and lysozyme to eliminate pathogenic microorganisms and maintain microbiota homeostasis, while enteroendocrine cells secrete GLP-1, 5-HT, and other regulators to modulate gastrointestinal motility, appetite, and glucose metabolism [27]. Regardless of formulation, protein supplementation significantly increased the number of secretory cells compared with the LPS group (*p* < 0.05). Among the blended-protein regimens, all three LF–OPN combinations resulted in a significantly greater abundance of secretory cells than OPN monotherapy (*p* < 0.05). Furthermore, the 5:1 and 10:1 LF-to-OPN ratios also yielded greater secretory-cell abundance than LF monotherapy (*p* < 0.05). In agreement with the present results, Hong and colleagues demonstrated that LF markedly elevated villus height, crypt depth, and the villus height-to-crypt depth ratio in a mouse model of intestinal injury, while also increasing goblet-cell numbers, promoting mucin secretion, enhancing the mucous barrier function, and reducing mucosal mechanical damage [28]. Huang et al. confirmed that exogenous bovine OPN supplementation significantly increased villus length, crypt depth, and the villus length/crypt depth ratio in intestinal injury model mice, while also increasing goblet-cell abundance, enhancing mucin secretion, strengthening mucus barrier function, and reducing the risk of bacterial translocation [29]. Collectively, the data indicate that LF and OPN each markedly attenuate gut-barrier damage triggered by LPS-induced intestinal injury. Furthermore, their co-administration appears to provide an enhanced protective effect on intestinal.

Inflammatory cytokines are key signaling molecules that regulate intestinal homeostasis, barrier integrity, mucosal repair, and immune balance. The dynamic regulation of cytokine expression is essential for maintaining intestinal physiological function, whereas persistently elevated levels of pro-inflammatory cytokines are important drivers of intestinal injury and disease progression [4,30]. Our results showed that LPS significantly downregulated *Il10* expression and significantly upregulated *Il1b*, *Il6*, and *Tnfa* expression levels (*p* < 0.05), consistent with the findings of Shi et al [20]. Kim et al. demonstrated in both in vivo and in vitro experiments that LF significantly downregulated pro-inflammatory cytokines, including *Il1b*, *Il6*, and *Tnfa*, in intestinal injury-model intestinal tissues, while upregulating the anti-inflammatory cytokine *Il10* [22]. Han et al. reported that OPN inhibits secretion of pro-inflammatory mediators *Il1b*, *Il6*, and *Tnfa*, yet enhances production of the anti-inflammatory cytokine *Il10*, thus attenuating intestinal inflammation [31]. Consistent with these previous reports, all protein-treatment groups exhibited significantly higher intestinal *Il10* mRNA levels than the LPS group (*p* < 0.05), while pro-inflammatory factors such as *Il1b*, IL-6, and *Tnfa* were concomitantly suppressed (*p* < 0.05). In particular, all LF–OPN combination regimen upregulated *Il10* more effectively than LF monotherapy (*p* < 0.05), while also suppressing *Tnfa* to a greater extent than either single-protein treatment (*p* < 0.05). These findings suggest that LF–OPN co-administration exerts an enhanced anti-inflammatory effect in the LPS-induced intestinal injury model.

In the present study, *Tlr4* and *Myd88* were measured only at the transcriptional level as LPS-responsive inflammatory markers. Although the observed reduction in *Tlr4* and *Myd88* transcript levels is consistent with attenuation of LPS-associated inflammatory signaling, these findings do not establish direct inhibition of TLR4 signaling or demonstrate that TLR4 serves as a receptor for LF or OPN. Therefore, future experiments will be required to clarify the upstream mechanism and determine whether a true biological combined exists.

## 5. Conclusions

In summary, the LF and OPN composite protein significantly attenuated LPS-induced macrophage inflammatory responses, markedly alleviated intestinal-barrier damage caused by LPS-induced intestinal injury, and considerably reduced both local intestinal and systemic inflammatory cytokine levels. Notably, multiple indicators in the composite protein intervention groups were significantly superior to those in LF group and OPN group, indicating a combined effect between LF and OPN. However, the underlying mechanisms of their combined anti-inflammatory action and the specific pathways through which LF and OPN achieve this warrant further investigation.

## Figures and Tables

**Figure 1:**
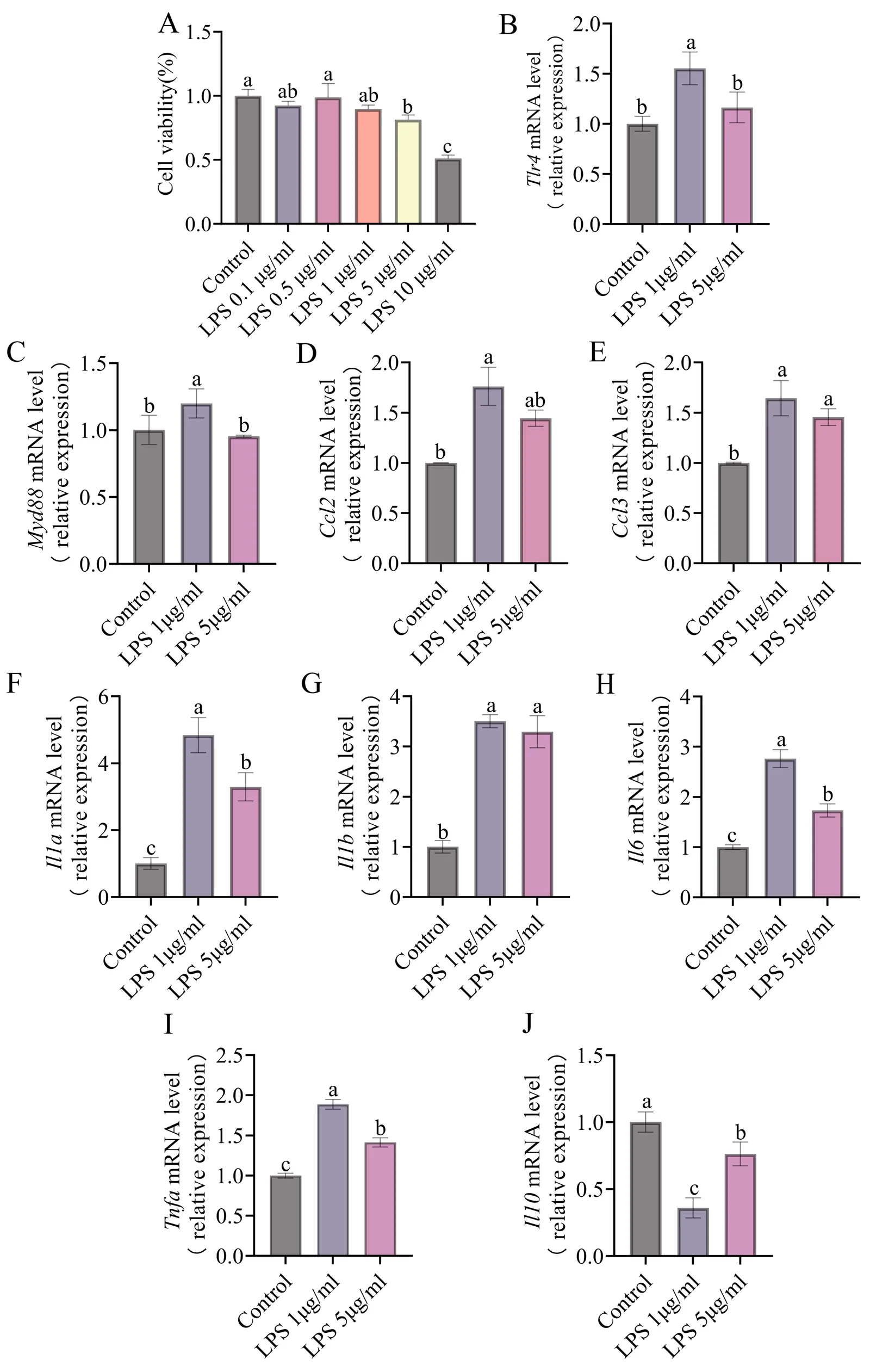
Effects of LPS on macrophage viability and inflammatory cytokine expression. (**A**) Macrophage viability after treatment with various LPS concentrations. (**B**–**J**) mRNA expression of *Tlr4* (**B**), *Myd88* (**C**), *Ccl2* (**D**), *Ccl3* (**E**), *Il1a* (**F**), *Il1b* (**G**), *Il6* (**H**), *Tnfa* (**I**), and *Il10* (**J**) in macrophages exposed to different LPS doses. All measurements are presented as mean ± SD. Differences across experimental groups were evaluated using one-way ANOVA with Tukey’s post hoc test. Statistically significant differences are marked by unique lowercase letters atop each column (*p* < 0.05), whereas columns bearing the same letter are not statistically different (*p* > 0.05).

**Figure 2:**
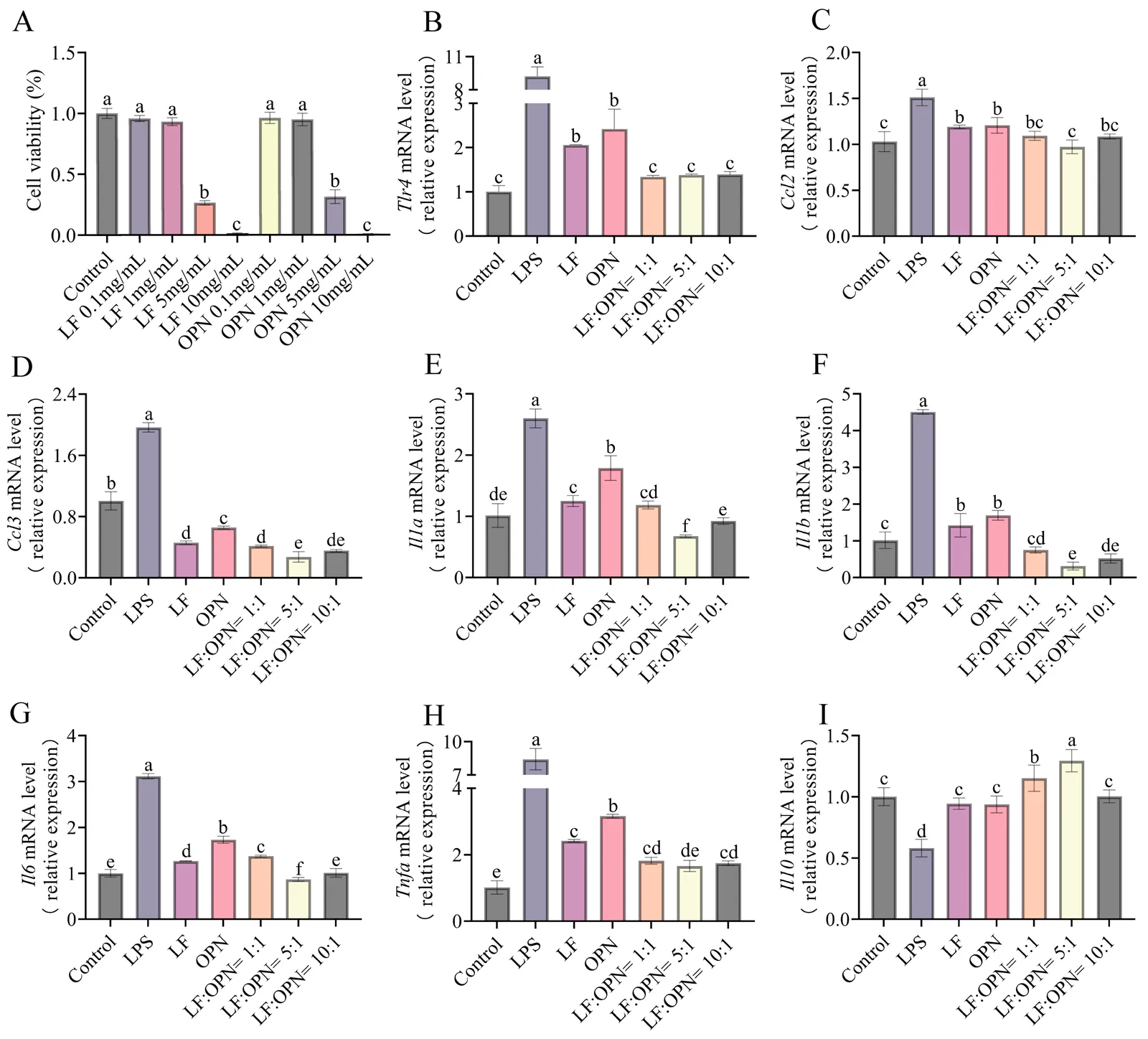
LF and OPN dosages and proportions modulate macrophage survival and pro-inflammatory mediator-transcript abundance. (**A**) Macrophage survival after exposure to escalating doses of LF or OPN. (**B**–**I**) Transcript abundance of *Tlr4* (**B**), *Ccl2* (**C**), *Ccl3* (**D**), *Il1a* (**E**), *Il1b* (**F**), *Il6* (**G**), *Tnfa* (**H**), and *Il10* (**I**) in macrophages subjected to various protein interventions. Data are expressed as mean ± SD. Intergroup variance was evaluated through one-way ANOVA and Tukey’s post hoc comparison. Bars annotated with dissimilar lowercase letters are significantly different (*p* < 0.05), whereas columns with matching annotations are not significantly different (*p* > 0.05).

**Figure 3:**
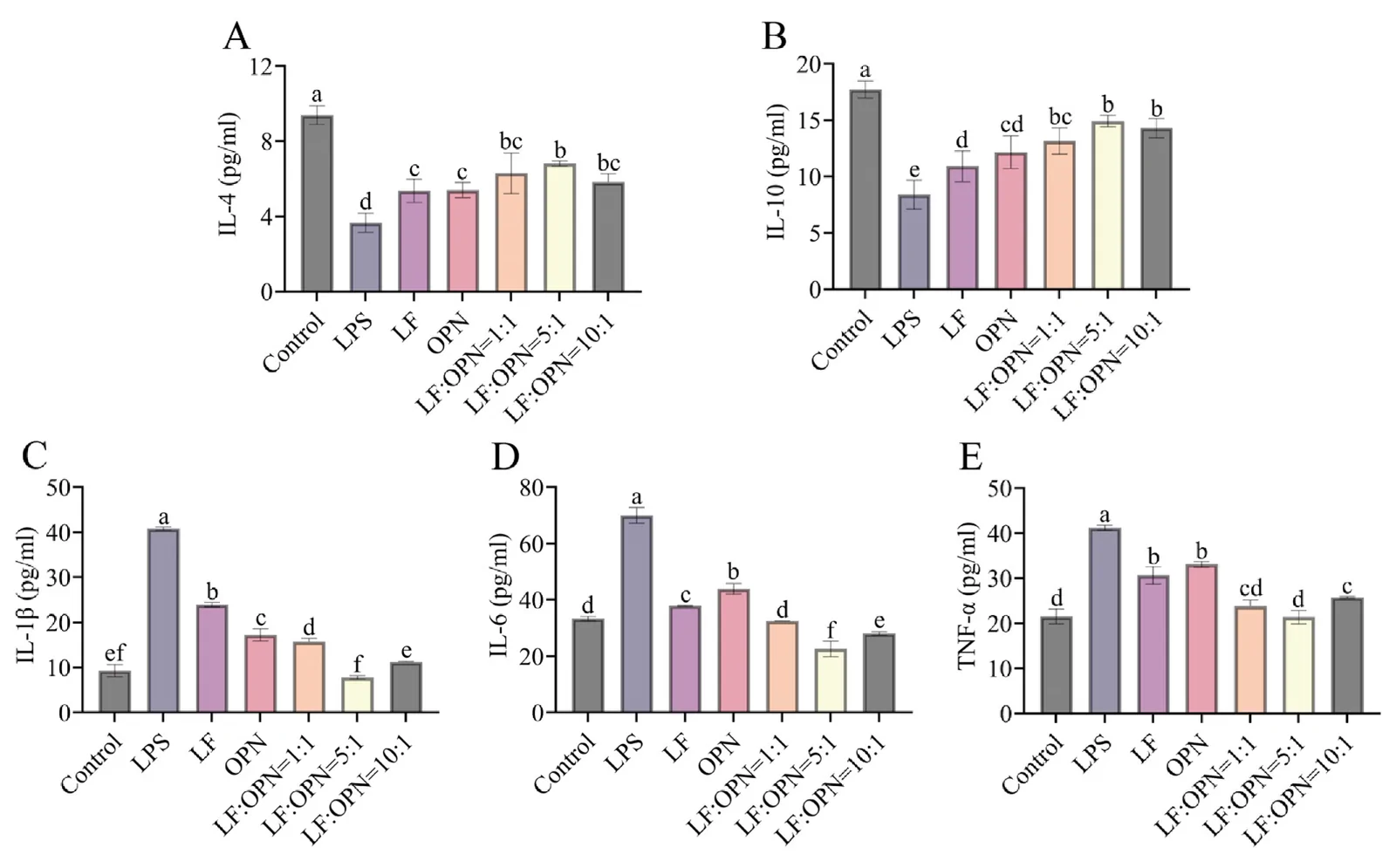
Different LF and OPN combinations alter inflammatory-factor protein levels within macrophages. (**A**–**E**) Protein expression levels of IL-4, IL-10, IL-1β, IL-6, and TNF-α in macrophages following various protein interventions, corresponding to panels (**A**) through (**E**), respectively. All measurements are reported as mean ± standard deviation. Statistical differences among groups were determined by one-way ANOVA followed by Tukey’s post hoc test. Bearing different lowercase letters denotes statistical significance between groups (*p* < 0.05), whereas a shared letter indicates a lack of significant difference (*p* > 0.05).

**Figure 4:**
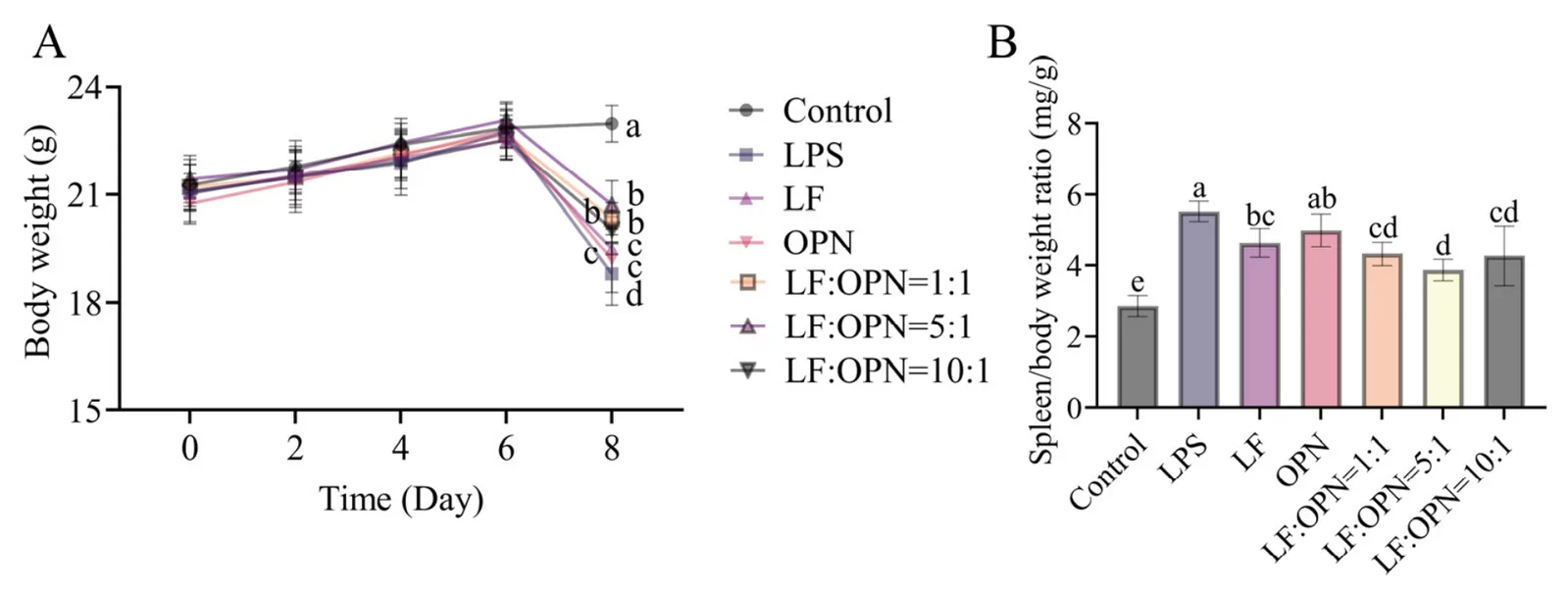
Different LF and OPN combinations influence body mass and Splenic Index in a murine model. (**A**) Temporal alterations in body mass among experimental groups throughout the treatment phase. (**B**) Spleen-to-body weight ratios for each experimental cohort. Data points represent mean ± standard deviation, with six animals per group (*n* = 6). One-way ANOVA followed by Tukey’s post hoc procedure was utilized to perform cross-group statistical testing. Different lowercase letters above columns mark statistically significant disparities (*p* < 0.05), whereas matching letters signify the absence of statistical significance (*p* > 0.05).

**Figure 5:**
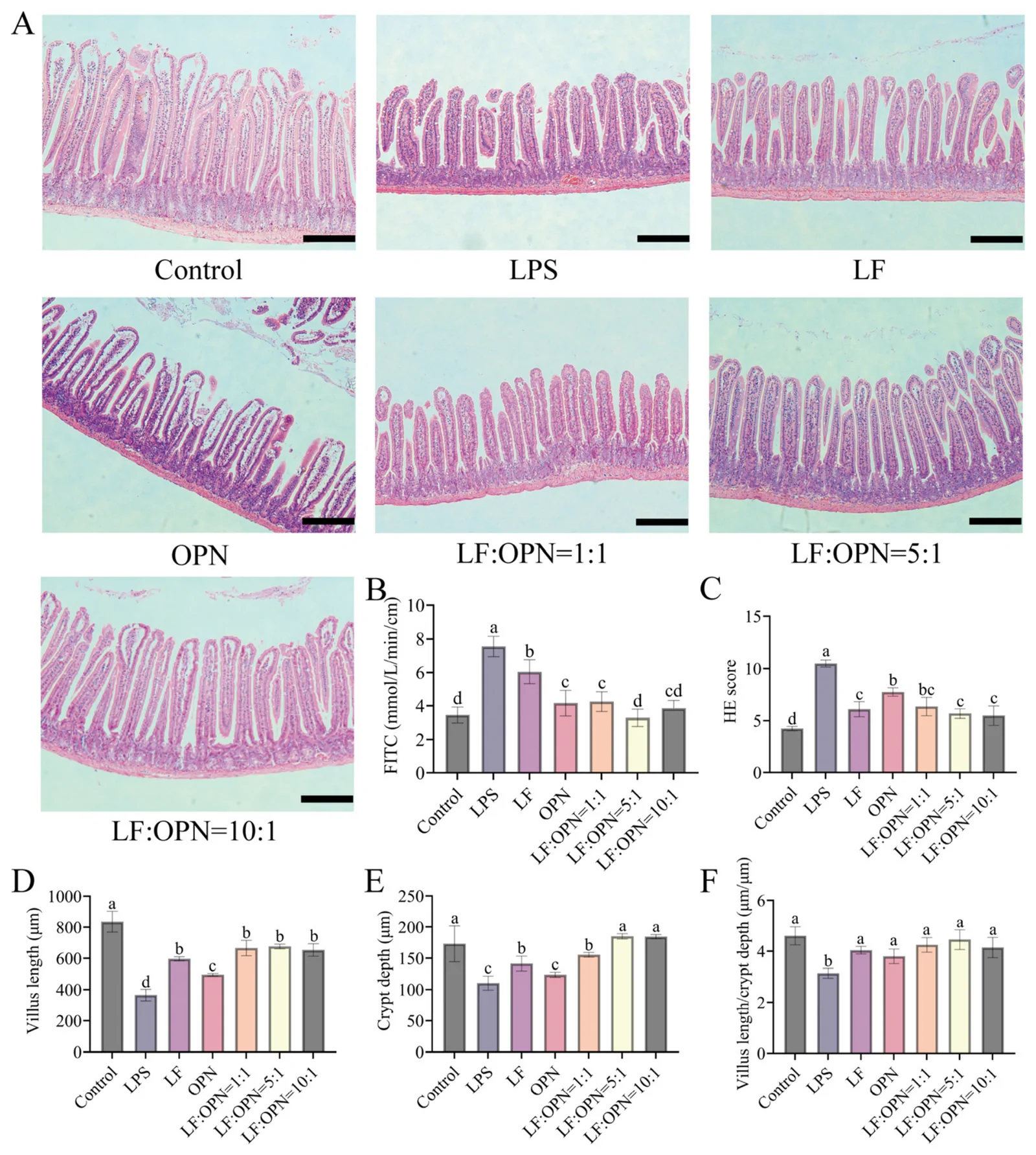
Different LF and OPN combinations modulate gut-barrier permeability and mucosal architecture in a murine model. (**A**) Representative HE-stained intestinal sections; (**B**) gut permeability (scale bar = 200 μm); (**C**) histopathological (HE) scores; (**D**) villus height; (**E**) crypt depth; and (**F**) villus-to-crypt ratio. All parameters were assessed in intestinal tissue from each experimental group. Numerical data are expressed as mean ± standard deviation, with *n* = 6 animals per group. Statistical differences across groups were evaluated using one-way ANOVA and Tukey’s post hoc analysis. Distinct lowercase letters assigned to columns indicate statistically significant differences (*p* < 0.05), whereas a common letter indicates the absence of statistical significance (*p* > 0.05).

**Figure 6:**
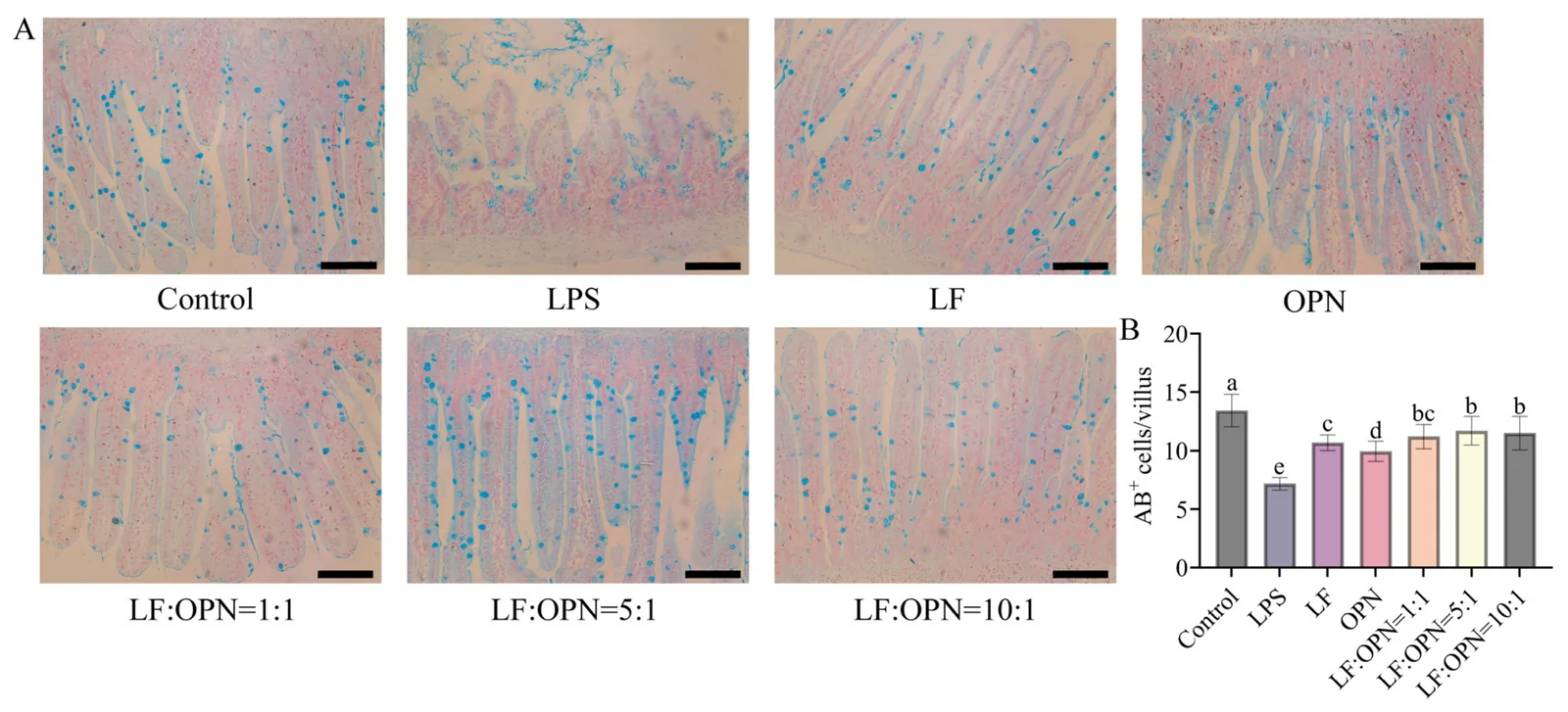
Different LF and OPN combinations enhance goblet-cell enumeration in murine intestinal tissue. (**A**) Representative Alcian Blue-stained intestinal sections (scale bar = 100 μm). (**B**) Enumeration of goblet cells per experimental group. Results are displayed as mean ± standard deviation, with six replicates per group (*n* = 6). One-way ANOVA coupled with Tukey’s post hoc test was adopted for intergroup statistical analysis. Unique lowercase letters atop each column mark significant disparities (*p* < 0.05), whereas matching letter assignments correspond to non-significant comparisons (*p* > 0.05).

**Figure 7:**
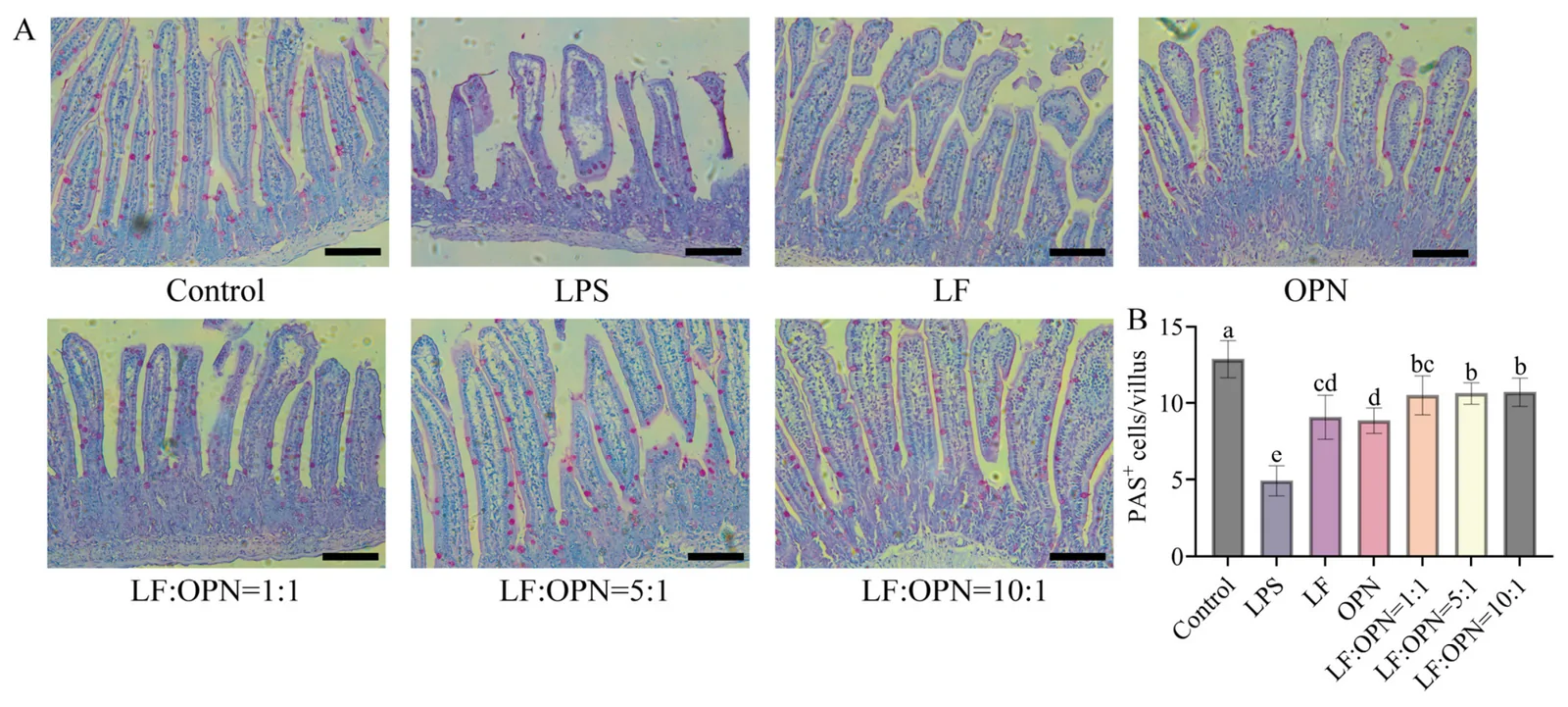
Different LF and OPN combinations boost secretory-cell abundance in murine gut mucosa. (**A**) Representative PAS-stained gut tissue micrographs (scale bar = 100 μm). (**B**) Morphometric quantification of secretory cells across experimental groups. Values are presented as mean ± SD (six animals per group). Intergroup differences were assessed via one-way ANOVA and Tukey’s post hoc comparison. Bearing unique lowercase letters marks significant disparities (*p* < 0.05), whereas matching letters denote the absence of statistical significance (*p* > 0.05).

**Figure 8:**
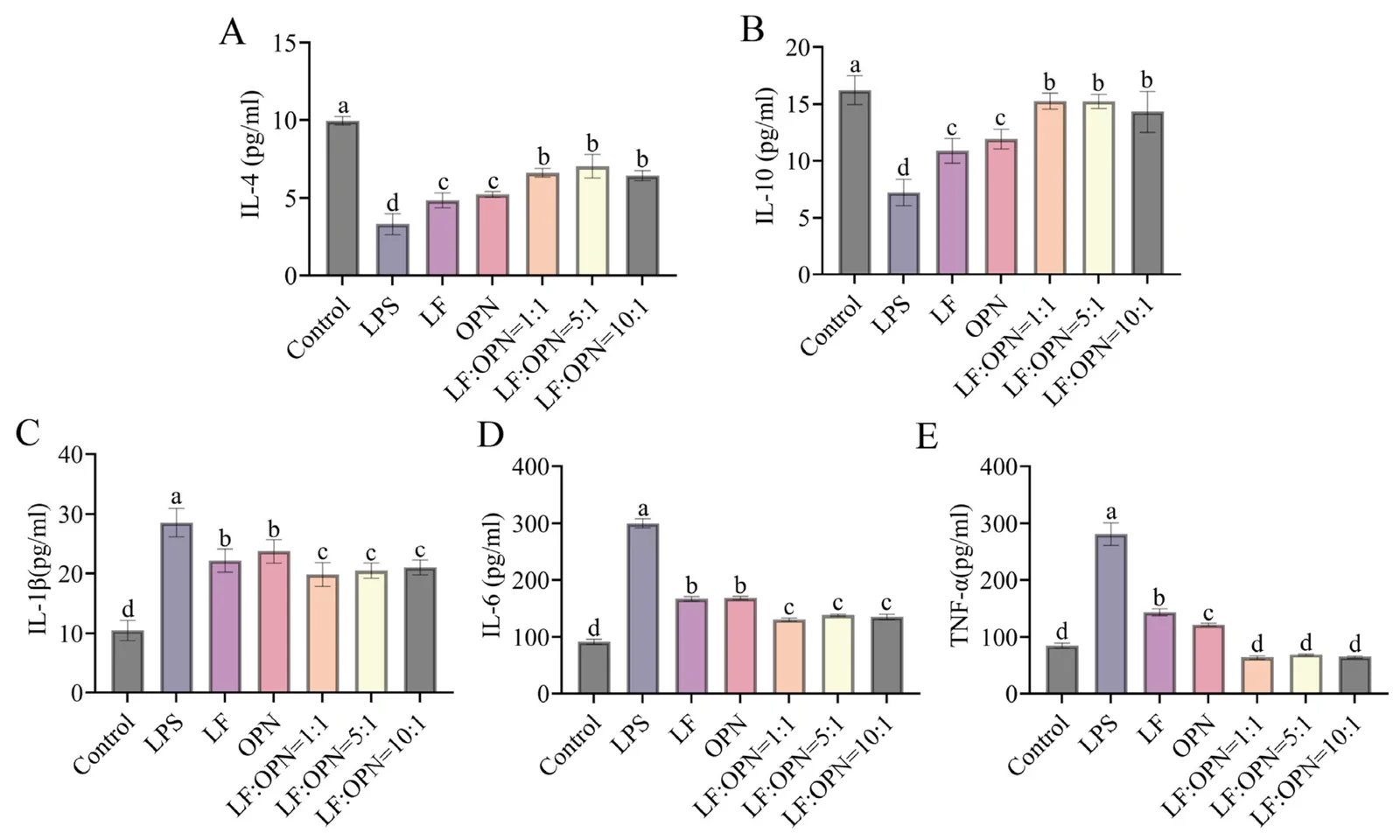
Different LF and OPN combinations attenuate circulating inflammatory mediator concentrations in a murine model. (**A**–**E**) Circulating protein levels of IL-4 (**A**), IL-10 (**B**), IL-1β (**C**), IL-6 (**D**), and TNF-α (**E**) in all experimental cohorts. Results are presented as mean ± SD, with six replicates per cohort (*n* = 6). Statistical comparisons across cohorts relied on one-way ANOVA with Tukey’s post hoc test. Distinct lowercase letters atop bars denote statistical significance (*p* < 0.05), while shared letters indicate non-significant outcomes (*p* > 0.05).

**Figure 9:**
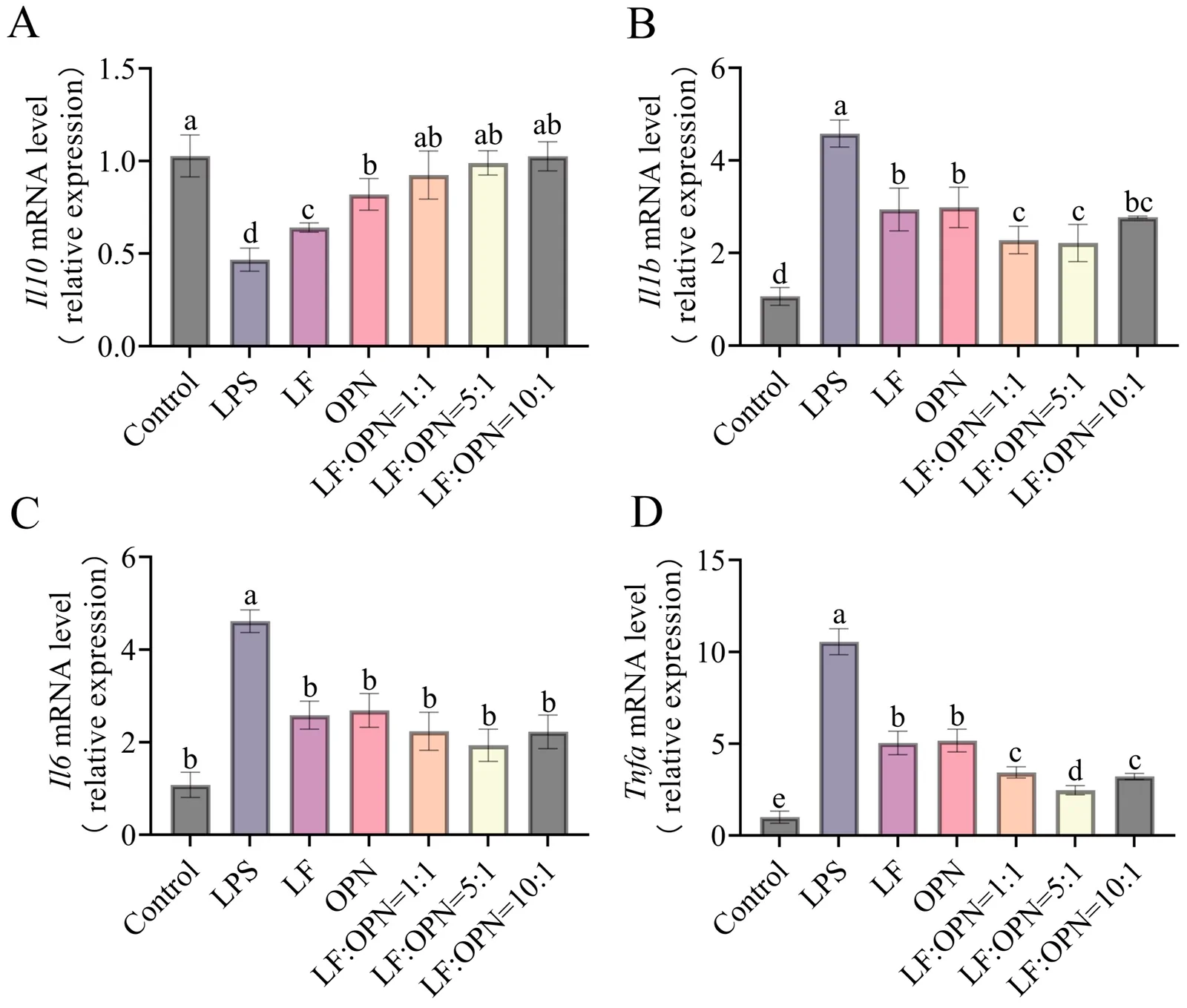
Different LF and OPN combinations dampen gut mucosal inflammatory mediator transcript levels within murine intestine. (**A**–**D**) Relative transcript abundance of *Il10* (**A**), *Il1b* (**B**), *Il6* (**C**), and *Tnfa* (**D**) in gut tissue from all experimental groups. Data are presented as mean ± SD (six animals per group). Statistical comparisons across groups relied on one-way ANOVA and Tukey’s post hoc analysis. Different lowercase letters atop columns indicate statistically significant disparities (*p* < 0.05), whereas columns with matching letters exhibit no significant difference (*p* > 0.05).

**Table 1:** Primer sequences used in RT-qPCR.

Gene	Forward Primer Sequence	Reverse Primer Sequence
*Tlr4*	AGCTTCTCCAATTTTTCAGAACTTC	TGAGAGGTGGTGTAAGCCATGC
*Myd88*	ACCTGTGTCTGGTCCATTGCCA	GCTGAGTGCAAACTTGGTCTGG
*Ccl2*	GCTACAAGAGGATCACCAGCAG	GTCTGGACCCATTCCTTCTTGG
*Ccl3*	ACTGCCTGCTGCTTCTCCTACA	ATGACACCTGGCTGGGAGCAAA
*Il4*	ATCATCGGCATTTTGAACGAGGTC	ACCTTGGAAGCCCTACAGACGA
*Il10*	CGGGAAGACAATAACTGCACCC	CGGTTAGCAGTATGTTGTCCAGC
*Il1a*	ACGGCTGAGTTTCAGTGAGACC	CACTCTGGTAGGTGTAAGGTGC
*Il1b*	TGGACCTTCCAGGATGAGGACA	GTTCATCTCGGAGCCTGTAGTG
*Il6*	TACCACTTCACAAGTCGGAGGC	CTGCAAGTGCATCATCGTTGTTC
*Tnfa*	AGCAAGTGCAGGAAAGCTGGCT	GCTTTCGCAGAGGCAGTAACAG

## Data Availability

The original contributions presented in this study are included in the article. Further inquiries can be directed to the corresponding authors.

## Abbreviations

The following abbreviations are used in this manuscript:

LF	lactoferrin
OPN	osteopontin
UC	ulcerative colitis
CD	Crohn’s disease
CRC	colorectal cancer
FITC	fluorescein isothiocyanate
RT-qPCR	real-time polymerase chain reaction
MyD88	Myeloid Differentiation Primary Response 88
CCL-2	C-C motif chemokine ligand 2
CCL-3	C-C motif chemokine ligand 3
IL-4	interleukin-4
IL-10	interleukin-10
IL-1α	interleukin-1α
IL-1β	interleukin-1β
TNF-α	Tumor Necrosis Factor α
GAPDH	glyceraldehyde-3-phosphate dehydrogenase
SD	standard deviation
ANOVA	analysis of variance
TGF-β	transforming growth factor-β
